# Modelling infectious diseases with relapse: a case study of HSV-2

**DOI:** 10.1186/s12976-017-0059-4

**Published:** 2017-07-17

**Authors:** Jinliang Wang, Xiaoqing Yu, Heidi L. Tessmer, Toshikazu Kuniya, Ryosuke Omori

**Affiliations:** 1 School of Mathematical Science, Heilongjiang University, Harbin, 150080 People’s Republic of China; 2 Division of Bioinformatics, Research Center for Zoonosis Control, Hokkaido University, Sapporo, Hokkaido, 001-0020 Japan; 3 Department of Applied Mathematics, Graduate School of System Informatics, Kobe University, 1-1 Rokkodai-cho, Nada-ku, Kobe, 657-8501 Japan; 4 JST, PRESTO, 4-1-8 Honcho, Kawaguchi, Saitama, 332-0012 Japan

**Keywords:** Multi-group SVIRI epidemic model, Relapse, Basic reproduction number, Global asymptotic stability, Herpes Simplex Virus Type 2, Vaccination

## Abstract

**Background:**

Herpes Simplex Virus Type 2 (HSV-2) is one of the most common sexually transmitted diseases. Although there is still no licensed vaccine for HSV-2, a theoretical investigation of the potential effects of a vaccine is considered important and has recently been conducted by several researchers. Although compartmental mathematical models were considered for each special case in the previous studies, as yet there are few global stability results.

**Results:**

In this paper, we formulate a multi-group SVIRI epidemic model for HSV-2, which enables us to consider the effects of vaccination, of waning vaccine immunity, and of infection relapse. Since the number of groups is arbitrary, our model can be applied to various structures such as risk, sex, and age group structures. For our model, we define the basic reproduction number ℜ_0_ and prove that if ℜ_0_≤1, then the disease-free equilibrium is globally asymptotically stable, whereas if ℜ_0_>1, then the endemic equilibrium is so. Based on this global stability result, we estimate ℜ_0_ for HSV-2 by applying our model to the risk group structure and using US data from 2001 to 2014. Through sensitivity analysis, we find that ℜ_0_ is approximately in the range of 2-3. Moreover, using the estimated parameters, we discuss the optimal vaccination strategy for the eradication of HSV-2.

**Conclusions:**

Through discussion of the optimal vaccination strategy, we come to the following conclusions. (1) Improving vaccine efficacy is more effective than increasing the number of vaccines. (2) Although the transmission risk in female individuals is higher than that in male individuals, distributing the available vaccines almost equally between female and male individuals is more effective than concentrating them within the female population.

## Background

Herpes Simplex Virus Type 2 (HSV-2) is one of the most common sexually transmitted diseases, and has infected about 417 million people aged 15-49 worldwide [1]. Although there is still no licensed vaccine for HSV-2, a theoretical investigation of the potential effects of a vaccine is considered important and has recently been conducted by several researchers (see [2–4]). In [2, 3], compartmental epidemic models with vaccination for HSV-2 were considered and the effectiveness of the vaccination was discussed in connection with the basic reproduction number ℜ_0_ (see [5]) through numerical simulations. However, there was little discussion about the stability of each equilibrium. As observed in several papers on epidemic models with vaccination (see, for instance, [6–8]), backward bifurcation can occur at ℜ_0_=1 for some special models and ℜ_0_<1 does not necessarily imply the global asymptotic stability of the disease-free equilibrium, that is, the eradication of the disease. In that case, the vaccination effort solely to make ℜ_0_<1 has less significance. Therefore, a global stability analysis is critical for theoretically justifying the epidemiological discussion.

In [4], Lou et al. considered a compartmental epidemic model for HSV-2 with age and risk group structures and discussed the effectiveness of the vaccination together with the global stability analysis of each equilibrium. In their study, the vaccination was limited to female individuals, who are known to be the high-risk group for HSV-2, and it was concluded that such a vaccination strategy can reduce the total infections in both females and males. However, to support their conclusion, we need to consider a more general model in which male individuals can also benefit from the vaccination and show that the optimal distribution ratio of the vaccines is 1 to 0 for female and male individuals. In this paper, we consider such a general model and investigate the optimal distribution ratio of the vaccines. As opposed to their conclusion, our result shows that distributing the vaccines almost equally to females and males is more effective for the eradication of HSV-2 than concentrating them within the female population.

To consider the effect of vaccination with imperfect immunity, SVIR epidemic models are often formulated, in which the total population is subdivided into the susceptible (*S*), vaccinated (*V*), infective (*I*) and recovered (*R*) populations (see, for instance, [2, 6–10]). However, to take into account the relapse of HSV-2 (see [2, 11]), it is necessary to also consider a direct transition from *R* to *I*. Thus, in this paper, we formulate a multi-group SVIRI epidemic model for HSV-2, which enables us to consider the effects of vaccination, of waning vaccine immunity, and of infection relapse. Since the number of groups is arbitrary, our model can be applied to various structures such as risk, sex, and age group structures. In the empirical portion of this paper, we apply our model to the risk group structure and estimate the basic reproduction number ℜ_0_ for HSV-2 by using data from the US from 2001 to 2014. Since the infective population of HSV-2 seems to be in endemic equilibrium, the estimation of ℜ_0_ must be carried out under the global asymptotic stability of the endemic equilibrium. However, in general, the global asymptotic stability of the endemic equilibrium is not trivial.

Recently, multi-group epidemic models have been studied by many authors [10, 12–24]. One of the most effective approaches for global stability analysis of multi-group epidemic models is the graph-theoretic approach developed by Guo et al. [14]. Since our model has a quite complex form with the paths from *V* to *S* (the waning of vaccine-induced immunity), *R* to *I* (relapse) and distributed time delay, the global asymptotic stability analysis is challenging. In this paper, by applying the graph-theoretic approach as in [14] together with an approach of max function as in [10], we prove that if ℜ_0_>1, then the endemic equilibrium is globally asymptotically stable, whereas if ℜ_0_≤1, then the disease-free equilibrium is so. Based on this theoretical result, we estimate ℜ_0_ for HSV-2 by using US data from 2001 to 2014. By using the estimated parameters, we discuss the optimal vaccination strategy for the eradication of HSV-2.

## Methods

### The general multi-group SVIRI epidemic model

Let $n \in \mathbb {N}$ be the number of groups and let ${\mathcal {N}} := \left \{ 1,2,\cdots, n \right \}$. Let *N*_*i*_(*t*) be the sexually active population in group $i \in {\mathcal {N}}$ at time *t*. Let us divide *N*_*i*_(*t*) into four subpopulations: susceptible *S*_*i*_(*t*), vaccinated *V*_*i*_(*t*), infective *I*_*i*_(*t*), and recovered *R*_*i*_(*t*). Thus, *N*_*i*_(*t*)=*S*_*i*_(*t*)+*V*_*i*_(*t*)+*I*_*i*_(*t*)+*R*_*i*_(*t*) for all $i \in {\mathcal {N}}$. We make the following assumptions: (A1) The number of individuals becoming sexually active in group $i \in {\mathcal {N}}$ per unit time is *b*_*i*_>0. (A2) The per capita rate of removal from the sexual activity in group $i \in {\mathcal {N}}$ is *μ*_*i*_>0. (A3) The coefficient for disease transmission from infective individuals in group $j \in {\mathcal {N}}$ to uninfected (susceptible or vaccinated) individuals in group $i \in {\mathcal {N}}$ is *β*_*ij*_≥0. The matrix $(\beta _{ij})_{i,j \in {\mathcal {N}}}$ is irreducible. The vaccine efficacy in group $i \in {\mathcal {N}}$ is *σ*_*i*_∈ [ 0,1] and the force of infection to vaccinated individuals in group $i \in {\mathcal {N}}$ is weakened by multiplying *σ*_*i*_. That is,

$$\begin{array}{@{}rcl@{}} {\lambda^{S}_{i}(t) :=} \sum_{j=1}^{n} \beta_{ij} \frac{I_{j} (t)}{N_{j}(t)} \ \ \text{and} \ \ {\lambda^{V}_{i}(t) :=} \sigma_{i} \sum_{j=1}^{n} \beta_{ij} \frac{I_{j} (t)}{N_{j}(t)}, \quad i \in {\mathcal{N}} \end{array} $$

are the forces of infection to susceptible and vaccinated individuals in group $i \in {\mathcal {N}}$ at time *t*≥0, respectively. Here we assume standard incidence. (A4) The per capita vaccination rate for susceptible individuals in group $i\in {\mathcal {N}}$ is *v*_*i*_>0. The per capita rate for the waning of vaccine-induced immunity for vaccinated individuals in group $i \in {\mathcal {N}}$ is *ω*_*i*_≥0. (A5) The per capita recovery rate of infective individuals in group $i \in {\mathcal {N}}$ is *γ*_*i*_>0. (A6) The survival probability for recovered individuals in group $i \in {\mathcal {N}}$, who spent time *t* in the recovered class, is $P_{i}(t):=\exp (-\int _{0}^{t} \delta _{i}(\eta) \mathrm {d}\eta)$, where *δ*_*i*_(*η*) denotes the relapse risk for individuals who spent time *η* in the recovered class in group *i*. For each $i \in {\mathcal {N}}$, $\delta _{i} \in L_{\text {loc}, +}^{1} (0,+\infty)$ and $\int _{0}^{+\infty }\delta _{i}(\eta)\mathrm {d}\eta = +\infty $.

Under assumptions (A1)-(A2), we see that the time variation of $N_{i}(t), \ i \in {\mathcal {N}}$ is governed by the following differential equation:

1 $$ N_{i}'(t) = b_{i} - \mu_{i} N_{i}(t), \ \ i \in {\mathcal{N}}.  $$

From the variation of constants formula, we easily see that ${\lim }_{t\to +\infty } N_{i}(t) = b_{i}/\mu _{i} =: N_{i}^{*}, \ i \in {\mathcal {N}}$. Hence, without loss of generality, we can assume that $N_{i}(t) \equiv N_{i}^{*}, \ i \in {\mathcal {N}}$. Then, under assumptions (A1)-(A4), we obtain the differential equations for *S*_*i*_(*t*) and *V*_*i*_(*t*), $i \in {\mathcal {N}}$ as follows:

2 $$\begin{array}{*{20}l} S_{i}'(t) =& b_{i} - S_{i}(t) \sum_{j=1}^{n} \beta_{ij} \frac{I_{j}(t)}{N_{j}^{*}} - \left(\mu_{i} + v_{i} \right) S_{i}(t) + \omega_{i} V_{i}(t),  \end{array} $$

3 $$\begin{array}{*{20}l} V_{i}'(t) =& v_{i} S_{i}(t) - \sigma_{i} V_{i}(t) \sum_{j=1}^{n} \beta_{ij} \frac{I_{j}(t)}{N_{j}^{*}} - \left(\mu_{i} + \omega_{i} \right) V_{i}(t).  \end{array} $$

Under assumptions (A5)-(A6), the recovered population in group $i \in {\mathcal {N}}$ at time *t* is given by

4 $$\begin{array}{*{20}l} R_{i} (t) =& \int_{0}^{+\infty} \gamma_{i} I_{i} (t-\xi) e^{-\mu_{i} \xi} e^{- \int_{0}^{\xi} \delta_{i}(\eta) \mathrm{d}\eta} \mathrm{d} \xi  \\ =& \int_{-\infty}^{t} \gamma_{i} I_{i} (\xi) e^{-\mu_{i} (t-\xi)} e^{- \int_{0}^{t-\xi} \delta_{i}(\eta) \mathrm{d}\eta} \mathrm{d} \xi, \quad i \in {\mathcal{N}}.  \end{array} $$

By differentiating (4), we obtain the following integro-differential equation for *R*_*i*_(*t*), $i \in {\mathcal {N}}$.

5 $$\begin{array}{*{20}l} R_{i}'(t) =& \gamma_{i} I_{i}(t) - \mu_{i} R_{i}(t) - \int_{-\infty}^{t} \delta_{i}(t-\xi) \gamma_{i} I_{i} (\xi) e^{-\mu_{i} (t-\xi)} e^{- \int_{0}^{t-\xi} \delta_{i}(\eta) \mathrm{d}\eta} \mathrm{d} \xi  \\ =& \gamma_{i} I_{i}(t) - \mu_{i} R_{i}(t) - \int_{0}^{+\infty} \delta_{i}(\xi) \gamma_{i} I_{i} (t-\xi) e^{-\mu_{i} \xi} e^{- \int_{0}^{\xi} \delta_{i}(\eta) \mathrm{d}\eta} \mathrm{d} \xi.  \end{array} $$

From (1)-(5) we obtain the integro-differential equation for *I*_*i*_(*t*), $i \in {\mathcal {N}}$ as follows.

$$\begin{array}{*{20}l} I_{i}'(t) =& \left(S_{i}(t) + \sigma_{i} V_{i}(t) \right) \sum_{j=1}^{n} \beta_{ij} \frac{I_{j}(t)}{N_{j}^{*}} - (\mu_{i} + \gamma_{i}) I_{i}(t)  \\ &+ \int_{0}^{+\infty} \delta_{i} (\xi) \gamma_{i} I_{i} (t-\xi) e^{-\mu_{i} \xi} e^{- \int_{0}^{\xi} \delta_{i}(\eta) \mathrm{d}\eta} \mathrm{d} \xi.  \end{array} $$

Under this setting, we arrive at the following main model in this paper.

6 $$ \left\{ \begin{aligned} S'_{i}(t) &=b_{i}-S_{i}(t) \sum\limits_{j=1}^{n} \beta_{ij}\frac{I_{j}(t)}{N_{j}^{*}} - (\mu_{i}+v_{i}) S_{i}(t) + \omega_{i} V_{i}(t),\\ V'_{i}(t) &=v_{i} S_{i}(t) - \sigma_{i} V_{i}(t) \sum\limits_{j=1}^{n}\beta_{ij} \frac{I_{j}(t)}{N_{j}^{*}} - \left(\mu_{i} + \omega_{i} \right) V_{i}(t),\\ I'_{i}(t) &= (S_{i}(t) + \sigma_{i} V_{i}(t))\sum\limits_{j=1}^{n}\beta_{ij} \frac{I_{j}(t)}{N_{j}^{*}} - (\mu_{i}+\gamma_{i}) I_{i}(t) \\ &\quad +\int_{0}^{+\infty} \delta_{i}(\xi) \gamma_{i} I_{i}(t-\xi)e^{-\mu_{i} \xi} e^{-\int_{0}^{\xi} \delta_{i}(\eta) \mathrm{d}\eta} \mathrm{d} \xi, \quad i \in {\mathcal{N}}. \end{aligned}\right.  $$

Note that the differential equation of $R_{i}(t), \ i \in {\mathcal {N}}$ can be omitted since it does not appear in the above three equations.

The equilibria of system (6) can be obtained as the solution of the following algebraic equations.

7 $$ \left\{ \begin{array}{ll} 0=b_{i}-S_{i} \sum\limits_{j=1}^{n} \beta_{ij}\frac{I_{j}}{N_{j}^{*}}-(\mu_{i}+v_{i}) S_{i} + \omega_{i} V_{i},\\ 0=v_{i} S_{i} -\sigma_{i} V_{i} \sum\limits_{j=1}^{n} \beta_{ij}\frac{I_{j}}{N_{j}^{*}} -\left(\mu_{i} + \omega_{i} \right) V_{i},\\ 0=(S_{i}+\sigma_{i}V_{i})\sum\limits_{j=1}^{n} \beta_{ij} \frac{I_{j}}{N_{j}^{*}} - (\mu_{i} +\gamma_{i} - Q_{i}) I_{i}, \quad i \in {\mathcal{N}}, \end{array}\right.  $$

where

$$Q_{i} := \int_{0}^{+\infty} \delta_{i}(\xi) \gamma_{i} e^{-\mu_{i} \xi} e^{-\int_{0}^{\xi} \delta_{i}(\eta) \mathrm{d}\eta} \mathrm{d}\xi, \quad i \in {\mathcal{N}}. $$

Note that

$$\begin{array}{*{20}l} Q_{i} <& \gamma_{i} \int_{0}^{+\infty} \delta_{i}(\xi) e^{-\int_{0}^{\xi} \delta_{i}(\eta) \mathrm{d}\eta} \mathrm{d}\xi = \gamma_{i} \left[ -e^{-\int_{0}^{\zeta} \delta_{i}(\eta) \mathrm{d}\eta} \right]_{0}^{+\infty} = \gamma_{i}, \quad i \in {\mathcal{N}}. \end{array} $$

Hence, we have *μ*_*i*_+*γ*_*i*_−*Q*_*i*_>0 for all $i \in {\mathcal {N}}$.

It is easy to see that the trivial solution of (7) such that *I*_*i*_=0 for all $i \in {\mathcal {N}}$ always exists. It is called the disease-free equilibrium of system (6) and we write it as $E^{0} := \left (S_{1}^{0}, V_{1}^{0}, 0, S_{2}^{0}, V_{2}^{0}, 0, \cdots, S_{n}^{0}, V_{n}^{0}, 0 \right) \in \mathbb {R}_{+}^{3n}$, where

$$S_{i}^{0} := \frac{b_{i}}{\mu_{i}} \frac{\mu_{i} + \omega_{i}}{\mu_{i} + v_{i} + \omega_{i}}, \quad V_{i}^{0} := \frac{v_{i}}{\mu_{i} + \omega_{i}} S_{i}^{0} = \frac{b_{i}}{\mu_{i}} \frac{v_{i}}{\mu_{i} + v_{i}+\omega_{i}}, \quad i \in {\mathcal{N}}. $$

Existence of the endemic equilibrium $E^{*} : = \left (S_{1}^{*}, V_{1}^{*}, I_{1}^{*}, \cdots, S_{n}^{*}, V_{n}^{*}, I_{n}^{*} \right) \in \mathbb {R}_{+}^{3n}$ such that $I_{i}^{*} > 0$ for all $i \in {\mathcal {N}}$ will be discussed in connection with the basic reproduction number ℜ_0_, which is defined as the expected number of secondary cases produced by a typical infected individual during its entire period of infectiousness at the initial invasion phase into a fully susceptible population, and given by the spectral radius of the next generation matrix (see [25]). Let

$$\mathcal{F}:=\left(\begin{array}{ccc} \left(S_{1}^{0}+\sigma_{1}v_{1}^{0}\right) \frac{\beta_{11}}{N_{1}^{*}} & \cdots & \left(S_{1}^{0}+\sigma_{1}v_{1}^{0}\right) \frac{\beta_{1n}}{N_{n}^{*}} \\ \vdots & \ddots & \vdots\\ \left(S_{n}^{0}+\sigma_{n}v_{n}^{0}\right) \frac{\beta_{n1}}{N_{1}^{*}} & \cdots & \left(S_{n}^{0}+\sigma_{n}v_{n}^{0}\right) \frac{\beta_{nn}}{N_{n}^{*}} \\ \end{array} \right) \text{and} \ \ \mathcal{V}:=\mathop{\text{diag}}_{1 \leq i \leq n} \left(\mu_{i}+\gamma_{i}-Q_{i} \right). $$

Then, according to [25], the next generation matrix is given by

$$\mathcal{K} := \mathcal{F}\mathcal{V}^{-1}=\left(\begin{array}{ccc} \frac{\left(S_{1}^{0}+\sigma_{1}V_{1}^{0}\right)\beta_{11}}{\left(\mu_{1}+\gamma_{1}-Q_{1}\right) N_{1}^{*}} & \cdots & \frac{\left(S_{1}^{0}+\sigma_{1}V_{1}^{0}\right)\beta_{1n}}{ \left(\mu_{n}+\gamma_{n}-Q_{n}\right) N_{n}^{*}} \\ \vdots & \ddots & \vdots\\ \frac{\left(S_{n}^{0}+\sigma_{n}V_{n}^{0}\right) \beta_{n1}}{\left(\mu_{1}+\gamma_{1}-Q_{1}\right) N_{1}^{*}} & \cdots & \frac{\left(S_{n}^{0}+\sigma_{n}V_{n}^{0}\right)\beta_{nn}}{\left(\mu_{n}+\gamma_{n}-Q_{n}\right) N_{n}^{*}} \\ \end{array} \right). $$

Hence, the basic reproduction number ℜ_0_ is defined by

8 $$ \Re_{0} := \rho (\mathcal{K}),  $$

where *ρ*(·) denotes the spectral radius of a matrix. We will obtain the global stability results for (6) in connection with ℜ_0_ (see the “Results” section).

### The special multi-group SVIRI epidemic model for HSV-2

The general model (6) can be applied to analyze the field data of HSV-2 epidemics. Similar to other sexually transmitted infections, the risk factor for HSV-2 infection is sexual behavior. To describe the heterogeneity of HSV-2 infection risk between host individuals, we characterize the group as the combination of sex and their sexual behavior. We consider the following levels of sexual activity: *x*=0,1,2,⋯,5 meaning the number of sexual partners within the last 12 months, where *x*=5 implies the number of sexual partners is 5 or more. Let *y*∈{1,2} denote the sex, 1 denotes male and 2 denotes female. Then, the risk group is characterized by *i*∈{1,2,⋯,12} in the following way: *i*=2*x*_*i*_+*y*_*i*_, where

$$(x_{i}, y_{i}) = \left\{ \begin{array}{l} \left(m-1,\ 1\right) \ \ \text{if} \ \ i= 2m -1, \\ \left(m-1,\ 2\right) \ \ \text{if} \ \ i=2m, \end{array} \right. \ m =1,2,\cdots, 6. $$

For example, *i*=2 corresponds to (*x*_*i*_,*y*_*i*_)=(0,2) and implies the group of female individuals with no sexual partners and *i*=11 corresponds to (*x*_*i*_,*y*_*i*_)=(5,1) and implies the group of male individuals with 5 or more sexual partners. Then, (6) can be written as follows:

9 $$ \left\{ \begin{aligned} &S'_{i}(t) = b_{i}-S_{i}(t)\sum\limits_{j=1}^{12} \beta_{ij} \frac{I_{j}(t)}{N_{j}^{*}}-(\mu_{i}+v_{i}) S_{i}(t) + \omega_{i} V_{i}(t),\\ &V'_{i}(t) = v_{i}S_{i}(t)-\sigma_{i}V_{i}(t)\sum\limits_{j=1}^{12} \beta_{ij}\frac{I_{j}(t)}{N_{j}^{*}}-\left(\mu_{i} + \omega_{i} \right) V_{i}(t),\\ &I'_{i}(t) = (S_{i}(t)+\sigma_{i} V_{i}(t))\sum\limits_{j=1}^{12} \beta_{ij}\frac{I_{j}(t)}{N_{j}^{*}}-(\mu_{i}+\gamma_{i}) I_{i}(t) \\ &\qquad\quad +\int_{0}^{+\infty} \delta_{i}(\xi) \gamma_{i} I_{i}(t-\xi)e^{-\mu_{i} \xi} e^{-\int_{0}^{\xi} \delta_{i}(\eta) \mathrm{d}\eta} \mathrm{d} \xi, \\ &i \in \{ 1,2,\cdots, 12\}. \end{aligned}\right.  $$

Note that (9) is a special case of (6). In this section, we assume that *δ*_*i*_(*ξ*)≡*δ*_*i*_>0 for all *i*∈{1,2,⋯,12}. Note that the assumption (A6) is satisfied. In this case, we have:

$$\begin{array}{*{20}l} \int_{0}^{+\infty} \delta_{i}(\xi) \gamma_{i} I_{i} (t-\xi) e^{-\mu_{i} \xi} e^{-\int_{0}^{\xi} \delta_{i}(\eta)\mathrm{d}\eta} \mathrm{d}\xi =& \delta_{i} \int_{0}^{+\infty} \gamma_{i} I_{i} (t-\xi) e^{-\mu_{i} \xi} e^{-\delta_{i} \xi} \mathrm{d}\xi \\ =& \delta_{i} R_{i}(t), \quad i \in \{ 1,2,\cdots, 12 \}. \end{array} $$

Hence, together with the Eq. 5 of *R*_*i*_(*t*), (9) can be simplified to the following multi-group SVIRI epidemic model.

10 $$ \left\{ \begin{aligned} S'_{i}(t) &= b_{i}-S_{i}(t)\sum\limits_{j=1}^{12} \beta_{ij} \frac{I_{j}(t)}{N_{j}^{*}}-(\mu_{i}+v_{i}) S_{i}(t) + \omega_{i} V_{i}(t),\\ V'_{i}(t) &= v_{i}S_{i}(t)-\sigma_{i}V_{i}(t)\sum\limits_{j=1}^{12} \beta_{ij}\frac{I_{j}(t)}{N_{j}^{*}}-\left(\mu_{i} + \omega_{i} \right) V_{i}(t),\\ I'_{i}(t) &= (S_{i}(t)+\sigma_{i} V_{i}(t))\sum\limits_{j=1}^{12} \beta_{ij}\frac{I_{j}(t)}{N_{j}^{*}}-(\mu_{i}+\gamma_{i}) I_{i}(t) + \delta_{i} R_{i}(t), \\ R_{i}'(t) &= \gamma_{i} I_{i}(t) - \left(\mu_{i} + \delta_{i} \right) R_{i}(t), \quad i \in \{ 1,2,\cdots, 12\}. \end{aligned}\right.  $$

No vaccine against HSV-2 infection is currently available, so we ignore the vaccinated class *V*_*i*_, *i*∈{1,2,⋯,12} in the estimation of ℜ_0_. Then, (10) can be rewritten as follows.

11 $$ \left\{ \begin{aligned} S'_{i}(t)&=b_{i}-S_{i}(t) \sum\limits_{j=1}^{12} \frac{\beta_{ij}}{N_{j}^{*}} I_{j}(t) -\mu_{i} S_{i}(t), \\ I'_{i}(t)&= S_{i}(t) \sum\limits_{j=1}^{12} \frac{\beta_{ij}}{N_{j}^{*}}I_{j}(t) - (\mu_{i}+\gamma_{i}) I_{i}(t) +\delta_{i} R_{i}(t), \\ R_{i}'(t) &= \gamma_{i} I_{i}(t) - \left(\mu_{i} + \delta_{i} \right) R_{i}(t), \quad i \in \{ 1,2,\cdots, 12\}. \end{aligned}\right.  $$

The basic reproduction number ℜ_0_ for (11) is obtained as the spectral radius of the following matrix.

$$\left(\begin{array}{ccc} \frac{S_{1}^{0}}{\mu_{1}+\gamma_{1}-Q_{1}}\frac{\beta_{1,1}}{N_{1}^{*}} & \cdots &\frac{S_{1}^{0}}{\mu_{12}+\gamma_{12}-Q_{12}}\frac{\beta_{1,12}}{N_{12}^{*}} \\ \vdots & \ddots & \vdots\\ \frac{S_{12}^{0}}{\mu_{1}+\gamma_{1}-Q_{1}}\frac{\beta_{12,1}}{N_{1}^{*}} & \cdots &\frac{S_{12}^{0}}{\mu_{12}+\gamma_{12}-Q_{12}} \frac{\beta_{12,12}}{N_{12}^{*}} \end{array} \right), $$

where *Q*_*i*_=*δ*_*i*_ *γ*_*i*_/(*μ*_*i*_+*δ*_*i*_) and we write *β*_*ij*_ as *β*_*i*,*j*_ for improved readability.

Transmission rates between the risk groups *i* and *j*, *β*_*ij*_, depend on sexual behavior and sex. We modeled *β*_*ij*_ as follows;

12 $$ \beta_{ij} = \rho_{x_{i} y_{i}} \rho_{x_{j} y_{j}} \mathbf{R}_{x_{i} x_{j}} \mathbf{S}_{y_{i} y_{j}}.  $$

The meaning of each symbol for *β*_*ij*_ is as follows.

- $\rho _{x_{i} y_{i}}$ denotes the HSV-2 infection risk for the risk group *i*. The risk group is stratified by sex and the number of partners within the last 12 months, the risk group *i* denotes the individuals whose number of partners within the last 12 months is *x*_*i*_ and sex is *y*_*i*_. $\rho _{x_{i} y_{i}}$ is given by;
  $$\rho_{x_{i} y_{i}} = c_{y_{i}} (x_{i} + 1)^{\phi}. $$
  Here, similar to previous modelling studies of sexually transmitted infections, we modeled the relationship between infection risk and sexual behavior by a power law function [26].
- *c* denotes the sex specific HSV-2 transmission coefficient.
- *ϕ* denotes the exponent parameter describing the heterogeneity of the infection risk between different sexual behaviors.
- **R** denotes the mixing matrix between the risk groups defined by sexual behavior, *x*;
  $$\mathbf{R}_{x_{i} x_{j}} = q \delta_{x_{i} x_{j}} + (1-q) \frac{\sum_{y} \rho_{xy} N_{xy}}{\sum_{x} \sum_{y} \rho_{xy} N_{xy}}. $$
  This is the classical one-parameter ‘preferred mixing’ formulation, proposed by [27].
- *δ* denotes Kronecker’s delta.
- *q* denotes assortative coefficient. When *q*=0, the mixing between risk groups defined by sexual behavior is ‘proportionately mixing’, and the mixing is ‘fully assortative mixing’ when *q*=1.
- **S** denotes the mixing matrix between sexes;
  $$\mathbf{S} = \left(\begin{array}{cc} a & 1- a \\ 1-a & a \end{array} \right). $$
- *a* denotes the proportion of homosexual behavior.

We will use the special model (11) with transmission rate (12) to estimate the basic reproduction number ℜ_0_ for HSV-2 (see the “Results” section), and (10) with (12) to discuss the effectiveness of vaccination strategy (see the “Discussion” section).

## Results

### The main theorem

The main theorem of this paper is obtained for the general multi-group SVIRI epidemic model (6). Since (6) has an infinite time delay, we define the fading memory space (see, for instance, [28, 29]) as follows:

13 $$\begin{array}{*{20}l} C_{\Delta} :=& \left\{ \phi \in C((-\infty, 0]; \mathbb{R}_{+}): \phi (s)e^{\Delta s} \ \mathrm{is \ uniformly \ continuous \ on} (-\infty, 0], \right.  \\ & \left. \ \sup_{s \leq 0} |\phi(s)| e^{\Delta s} < +\infty \right\}, \end{array} $$

where *Δ* is a positive constant such that $0 < \Delta < \min _{i \in {\mathcal {N}}} \{ \mu _{i} \}$. Let us define the following state space for system (6):

14 $$\begin{array}{*{20}l} \Omega :=& \left\{ \left(\psi_{1}, \psi_{2}, \cdots, \psi_{n}, \tilde{\psi}_{1}, \tilde{\psi}_{2}, \cdots, \tilde{\psi}_{n}, \phi_{1}(\cdot), \phi_{2}(\cdot), \cdots, \phi_{n}(\cdot) \right) \in \mathbb{R}_{+}^{2n} \times C_{\Delta}^{n}: \right.  \\ & \left. 0 < \psi_{i} < S_{i}^{0}, \ 0 < \tilde{\psi}_{i} < V_{i}^{0}, \ \phi_{i}(0) > 0, \right.  \\ & \left. 0 < \psi_{i} + \tilde{\psi}_{i} + \phi_{i}(0) < \frac{b_{i}}{\mu_{i}}, \ \ i \in {\mathcal{N}} \right\}.  \end{array} $$

The following proposition is proved:

#### **Proposition 1**

*Ω* is positively invariant for system (6).

The main theorem of this paper is as follows.

#### **Theorem 1**

Let ℜ_0_ and *Ω* be defined by (8) and (14), respectively. Let $\bar {\Omega }$ denote the closure of *Ω*. *(i)* If ℜ_0_≤1, then the disease-free equilibrium $E^{0} \in \bar {\Omega }$ of system (6) is globally asymptotically stable in *Ω* and there exists no endemic equilibrium *E* ^∗^ in $\bar {\Omega }$. *(ii)* If ℜ_0_>1, then the system (6) has the unique endemic equilibrium *E* ^∗^ in *Ω* and it is globally asymptotically stable in *Ω*.

For the proofs of Proposition 1 and Theorem 1, see the Appendix.

Theorem 1 still works for (10) since it is a special case of (6). In particular, although (10) does not include the integrated time delay, to our knowledge, there is no previous study on the global asymptotic stability of the endemic equilibrium of model (10). From this viewpoint, our main theorem can be regarded as valuable for the empirical study in the subsequent sections.

### Estimation of ℜ_0_ for HSV-2

Based on Theorem 1, we estimate the basic reproduction number ℜ_0_ for HSV-2 in the US from 2001 to 2014. For the estimation of ℜ_0_, we use the special model (11) with transmission rate (12). Note that (11) corresponds to the case where *v*_*i*_=*σ*_*i*_=*ω*_*i*_=0 for all *i*∈{1,2,⋯,12}. Although the case where *v*_*i*_=0 for all *i*∈{1,2,⋯,12} is excluded under assumption (A4), it is easy to check in a completely similar way as in the Appendix that the global stability result similar to Theorem 1 holds.

Previous study derived the value of *δ*_*i*_ and *γ*_*i*_ from empirical data, *δ*_*i*_ and *γ*_*i*_ are parameterized based on [30], 1/*δ*_*i*_=78.5 days and 1/*γ*_*i*_=13 days for all *i*∈{1,2,⋯,12}. Here note that we can regard *μ*_*i*_ as the removal rate from our system, which is given by the sum of the sexual-inactivation rate and the mortality rate among those who are sexually active. We assume that the sexual life span is 50 years (15-65 years old) and parameterize the mortality rate by the national representative census data in the US [31], *μ*_*i*_=0.0231 per year for all *i*∈{1,2,⋯,12}. Based on the previous studies [32] and [33], we obtain the estimations *q*=0.3 and *a*=0.02, respectively (see Table 1).

**Table 1:** The model parameters and related estimates

Parameter	Meaning	Value	Reference
*δ*_*i*_ (*i*=1,2,⋯,12)	Relapse risk	1/78.5	[30]
*γ*_*i*_ (*i*=1,2,⋯,12)	Recovery rate	1/13	[30]
*μ*_*i*_ (*i*=1,2,⋯,12)	Rate of removal from sexual activity	0.0231	[31]
*q*	Assortative coefficient	0.3	[32]
*a*	Proportion of homosexual behavior	0.02	[33]
*c*_1_	Transmission coefficient for male	0.228	Estimated
*c*_2_	Transmission coefficient for female	1.78	Estimated
*ϕ*	Exponent parameter	0.700	Estimated
ℜ_0_	Basic reproduction number	2.07	Estimated

Using the observed data of sero-prevalence of HSV-2 in the US from 2001 to 2014 reported by [34], sex specific transmission coefficient *c* and the exponent parameter *ϕ* were estimated by maximum likelihood estimation. Since the antibody against HSV-2 infection (IgG) provides life-long immunity [35], we fitted *I*+*R* to the observed data of the number of sero-positive cases for the estimation of *c* and *ϕ*. To estimate *c* and *ϕ*, endemic equilibria of *I*_*i*_ and *R*_*i*_ were solved numerically with varied *c*_1_ and *c*_2_ and *ϕ*, and the set of *c*_1_ and *c*_2_ maximizing the likelihood function was explored. The likelihood function for *c*_1_ and *c*_2_ is given by

$$L(c_{1}, c_{2}, \phi) = \mathop{\Pi}_{T} \mathop{\Pi}_{i} \mathop{\text{pmf}} \left(\mathop{\text{bin}} \left(N^{\text{data}}_{i,T}, \ \frac{I_{i}^{*} (c_{i},c_{2},\phi) + R_{i}^{*} (c_{1}, c_{2},\phi)}{N_{i}^{*}} \right), P_{i,T}^{\text{data}} \right). $$

**Fig. 1:**
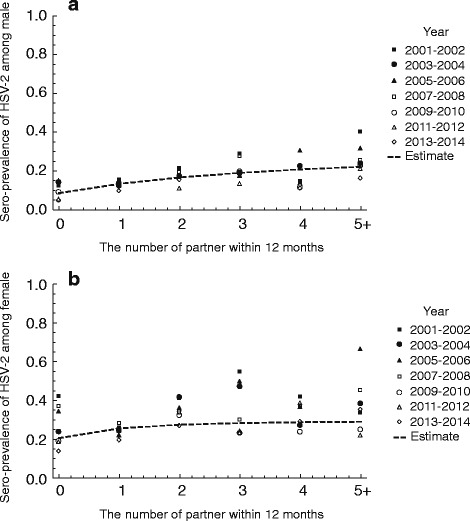
Comparison between the observed data of sero-prevalence of HSV-2 and the model estimates

Here pmf denotes the probability mass function, bin denotes a binomial distribution, $N^{\text {data}}_{i,T}$ denotes the observed data of the size of the risk group *i* in sampling year *T*, and $P^{\text {data}}_{i,T}$ denotes the observed data of the number of HSV2-seropositive cases in the risk group *i* in sampling year *T*, respectively. For the confidence interval (CI) of the estimated parameter, profile likelihood-based confidence intervals were calculated. Using estimated *c* and *ϕ* the basic reproduction number ℜ_0_ for HSV-2 in the US was calculated. Figure 1 shows the comparison between the observed data of sero-prevalence of HSV-2 and the model estimates. The estimated *c* are, transmission coefficient for male, *c*_1_=0.228 (95% CI 0.225 to 0.231), transmission coefficient for female, *c*_2_=1.78 (95% CI 1.75 to 1.81), exponent parameter *ϕ*=0.700 (95% CI 0.693 to 0.707) and estimated ℜ_0_=2.07 (95% CI 2.03 to 2.11).

**Fig. 2:**
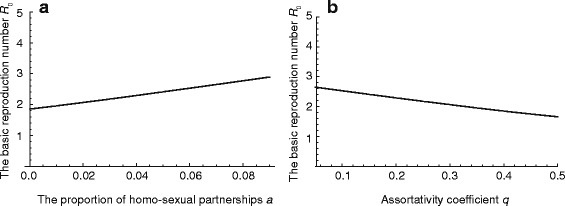
Sensitivity analysis with respect to the parameters describing sexual behavior *a* and *q*

Sexual behavior shows wide variation between host individuals. To assess the sensitivity of sexual behavior to ℜ_0_ of HSV-2, we conducted a sensitivity analysis of the parameters describing sexual behavior, i.e., the proportion of homosexual partnership *a* and assortativity coefficient for the mixing between risk groups *q*. Fig. 2 shows the relation of *a* and *q* to estimated ℜ_0_, ℜ_0_ increase if i) *a* increases, and ii) *q* decreases. The realistic variations of *a* and *q* [36, 37] can induce the variation of ℜ_0_, which is approximately demonstrated in the range of 2-3.

## Discussion

Using the demographic and epidemiological parameters obtained above, we discuss the effectiveness of each vaccination strategy. We investigate the sensitivity of the basic reproduction number ℜ_0_ to the vaccination parameters, that is, the vaccination rate among susceptible population *v* and the vaccination efficacy *σ*. Here we have assumed that vaccination is conducted with the same rate *v* for the susceptible population over time. For simplicity, we assume that the efficacy of vaccine *σ* is the same for all risk groups.

**Fig. 3:**
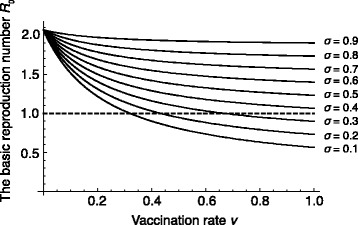
The relation of *v* to estimated ℜ_0_ with different *σ*

We first consider the case that vaccination rate *v* is the same between males and females. In this case, the basic reproduction number ℜ_0_ with different *σ* when *v* varies over (0,1) is shown in Fig. 3. We see from Fig. 3 that, if *σ* is 0.3 or smaller, ℜ_0_ can be less than 1. On the other hand, if *σ* is 0.4 or larger, ℜ_0_ cannot be less than 1 for any *v*∈(0,1). This implies that decreasing *σ* is more important than increasing *v* to reduce the basic reproduction number ℜ_0_. That is, improving the vaccine efficacy is more important for the eradication of HSV-2 than increasing the number of vaccines.

We next discuss the optimal sex ratio of the vaccinated population to control HSV-2. HSV-2 infection is observed among females more frequently than males, “opportunistic” vaccination can induce higher vaccination coverage among females than males. To assess the optimal sex ratio of the vaccination rate, we expand the vaccination rate *v* as follows;

$$v_{1} = p v, \quad v_{2} = (1-p)v, \quad v: \mathrm{total \ vaccination \ rate}. $$

**Fig. 4:**
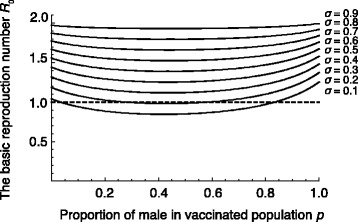
The relation of *p* and *σ* to estimated ℜ_0_

Here *p* denotes the sex ratio of vaccination. Figure 4 shows the relationship between *p*, *σ* and ℜ_0_, we assumed *v*=0.9 as the representative value. Interestingly, small or large *p* increases ℜ_0_. This implies that vaccination biased to females (small *p*) or males (large *p*) can result in persistence of the disease. In particular, it is noteworthy that the curves in Fig. 4 are almost symmetric with respect to *p* and the minimum is attained near the center *p*=0.5. This implies that vaccination distributed equally to females and males is optimal for the eradication of the disease even though the transmission coefficient for males is lower than that for females.

## Conclusion

In this paper, we have formulated the multi-group SVIRI epidemic model (6), which enables us to consider the effects of vaccination, the waning of vaccine-induced immunity, and relapse. We have defined the basic reproduction number ℜ_0_ and proved Theorem 1, which states that if ℜ_0_≤1, then the disease-free equilibrium *E* ^0^ is globally asymptotically stable, whereas if ℜ_0_>1, then the endemic equilibrium *E* ^∗^ is so. Based on Theorem 1, we have estimated the basic reproduction number ℜ_0_ for HSV-2 as 2.07 (95% CI 2.03 to 2.11) by using US HSV-2 data from 2001 to 2014. Through the sensitivity analysis for uncertain parameters on sexual behavior, we have found that ℜ_0_ is approximately in the range of 2-3. Furthermore, using sensitivity analysis for vaccination parameters, we have discussed the effectiveness of the vaccination. As a result, we have come to the following conclusions. (1) Improving vaccine efficacy is more effective than increasing the number of vaccines. (2) Although the transmission risk in female individuals is higher than that in male individuals, distributing vaccines almost equally to females and males is more effective than concentrating them within the female population.

## Appendix

### Proof of Proposition 1

We first show the positivity of the solution of system (6). Suppose that there exist *t*_1_>0 and $i^{*} \in {\mathcal {N}}$ such that *S*_*i*_(*t*)>0 and *V*_*i*_(*t*)>0 for all *t*∈[0,*t*_1_) and $i \in {\mathcal {N}}$ and $\min \left (S_{i^{*}}(t_{1}), V_{i^{*}}(t_{1}) \right) =0$. By the variation of constants formula, we have from the first equation in the system (6) that

$$\begin{array}{*{20}l} S_{i^{*}} (t_{1}) =& S_{i^{*}} (0) e^{-\int_{0}^{t_{1}} \left(\sum_{j=1}^{n} \beta_{i^{*}j} I_{j} (s)/N_{j}^{*} + \mu_{i^{*}} + v_{i^{*}} \right) \mathrm{d}s} \\ &+ \int_{0}^{t_{1}} \left(b_{i^{*}} + \omega_{i^{*}} V_{i^{*}}(s) \right) e^{-\int_{s}^{t_{1}} \left(\sum_{j=1}^{n} \beta_{i^{*}j} I_{j} (u)/N_{j}^{*} + \mu_{i^{*}} + v_{i^{*}} \right) \mathrm{d}u} \mathrm{d}s \ > 0. \end{array} $$

Hence, $V_{i^{*}}(t_{1}) = 0\phantom {\dot {i}\!}$. However, by the variation of constants formula, we have from the second equation in the system (6) that

$$\begin{array}{*{20}l} V_{i^{*}} (t) =& V_{i^{*}} (0) e^{-\int_{0}^{t_{1}} \left(\sigma_{i^{*}} \sum_{j=1}^{n} \beta_{i^{*}j} I_{j} (s)/N_{j}^{*} + \mu_{i^{*}} +\omega_{i^{*}} \right) \mathrm{d}s} \\ &+ \int_{0}^{t_{1}} v_{i^{*}} S_{i^{*}} (s) e^{-\int_{s}^{t_{1}} \left(\sigma_{i^{*}} \sum_{j=1}^{n} \beta_{i^{*}j} I_{j} (u)/N_{j}^{*} + \mu_{i^{*}} +\omega_{i^{*}} \right) \mathrm{d}u} \mathrm{d}s \ > 0, \end{array} $$

which is a contradiction. Hence, we see that *S*_*i*_(*t*)>0 and *V*_*i*_(*t*)>0 for all *t*>0 and $i \in {\mathcal {N}}$.

Suppose that there exist *t*_2_>0 and $\tilde {i} \in {\mathcal {N}}$ such that *I*_*i*_(*t*)>0 for all *t*∈[0,*t*_2_) and $i \in {\mathcal {N}}$ and $I_{\tilde {i}} (t_{2}) = 0$. By the variation of constants formula, we have from the third equation in the system (6) that

15 $$\begin{array}{*{20}l} I_{i} (t) =& I_{i} (0) e^{-(\mu_{i} + \gamma_{i}) t} +\int_{0}^{t} \left((S_{i} (s) + \sigma_{i} V_{i} (s)) \sum_{j=1}^{n} \beta_{ij} \frac{I_{j} (s)}{N_{j}^{*}} + h_{i}(s) \right) e^{-(\mu_{i} + \gamma_{i}) (t-s)} \mathrm{d}s,  \end{array} $$

where $h_{i} (t) := \int _{0}^{+\infty } \delta _{i}(\xi) \gamma _{i} I_{i}(t-\xi)e^{-\mu _{i} \xi } e^{-\int _{0}^{\xi } \delta _{i}(\eta) \mathrm {d}\eta } \mathrm {d}\xi $. We see that *h*_*i*_(*t*)≥0 for all $i \in {\mathcal {N}}$ and *t*∈ [ 0,*t*_1_). Hence, from (15), we have $I_{\tilde {i}}(t_{2}) > 0$, which is a contradiction. Hence, we see that *I*_*i*_(*t*)>0 for all *t*>0 and $i \in {\mathcal {N}}$.

The boundedness of the solution of system (6) immediately follows from the fact that *N* *i*′(*t*)=*b*_*i*_−*μ*_*i*_ *N*_*i*_(*t*), *S* *i*′(*t*)≤*b*_*i*_−(*μ*_*i*_+*v*_*i*_)*S*_*i*_(*t*)+*ω*_*i*_ *V*_*i*_(*t*) and *V* *i*′(*t*)≤*v*_*i*_ *S*_*i*_(*t*)−(*μ*_*i*_+*ω*_*i*_)*V*_*i*_(*t*) for all *t*>0 and $i \in {\mathcal {N}}$. This completes the proof.

### Proof of (i) of Theorem 1

We define the following matrix, which corresponds to the next generation matrix:

16 $$ M^{0} := \mathcal{V}^{-1}\mathcal{F} = \left(\begin{array}{ccc} \frac{\left(S_{1}^{0}+\sigma_{1}V_{1}^{0}\right)\beta_{11}}{\left(\mu_{1}+\gamma_{1}-Q_{1} \right) N_{1}^{*}} & \cdots & \frac{\left(S_{1}^{0}+\sigma_{1}V_{1}^{0}\right)\beta_{1n}}{\left(\mu_{1}+\gamma_{1}-Q_{1} \right) N_{n}^{*}} \\ \vdots & \ddots & \vdots\\ \frac{\left(S_{n}^{0}+\sigma_{n}V_{n}^{0}\right)\beta_{n1}}{\left(\mu_{n}+\gamma_{n}-Q_{n} \right) N_{1}^{*}} & \cdots & \frac{\left(S_{n}^{0}+\sigma_{n}V_{n}^{0}\right)\beta_{nn}}{\left(\mu_{n}+\gamma_{n}-Q_{n} \right) N_{n}^{*}} \end{array} \right).   $$

In fact, it is easy to see that $\rho (M^{0}) = \rho (\mathcal {K}) =\Re _{0}$.

First we show that system (6) has no endemic equilibrium $E^{*} \in \bar {\Omega }$. Let us define the following matrix-valued function on $\mathbb {R}^{2n}$, which is equal to *M* ^0^ if $(S_{1},V_{1},\cdots,S_{n},V_{n}) = (S^{0}_{1},V^{0}_{1}, \cdots S^{0}_{n}, V^{0}_{n})$:

$$M (S_{1}, V_{1}, \cdots, S_{n}, V_{n}) := \left(\begin{array}{ccc} \frac{(S_{1}+\sigma_{1}V_{1})\beta_{11}}{\left(\mu_{1}+\gamma_{1}-Q_{1} \right) N_{1}^{*}} & \cdots & \frac{(S_{1}+\sigma_{1}V_{1})\beta_{1n}}{\left(\mu_{1}+\gamma_{1}-Q_{1} \right) N_{n}^{*}} \\ \vdots & \ddots & \vdots\\ \frac{(S_{n}+\sigma_{n}V_{n})\beta_{n1}}{\left(\mu_{n}+\gamma_{n}-Q_{n} \right) N_{1}^{*}} & \cdots & \frac{(S_{n}+\sigma_{n}V_{n})\beta_{nn}}{\left(\mu_{n}+\gamma_{n}-Q_{n} \right) N_{n}^{*}} \end{array} \right). $$

Suppose that $(S_{1},\cdots,S_{n}) \neq (S_{1}^{0},\cdots, S_{n}^{0})$. Then, from assumptions (A1)-(A6), we see that **0**<*M*(*S*_1_,*V*_1_,⋯,*S*_*n*_,*V*_*n*_)<*M* ^0^, where **0** denotes the zero matrix and the inequality implies that it holds for each element and each of the two matrices are not equal. Then, since it follows from assumptions (A1)-(A6) that matrices *M* ^0^ and *M* ^0^+*M*(*S*_1_,*V*_1_,⋯,*S*_*n*_,*V*_*n*_) are nonnegative and irreducible, we can apply the Perron-Frobenius theorem (see [38, Corollary 2.1.5]) to obtain that *ρ*(*M*(*S*_1_,*V*_1_,⋯,*S*_*n*_,*V*_*n*_))<*ρ*(*M* ^0^)≤1. This implies that the equation *M*(*S*_1_,*V*_1_,⋯,*S*_*n*_,*V*_*n*_)(*I*_1_,⋯*I*_*n*_)^*T*^=(*I*_1_,⋯*I*_*n*_)^*T*^ has only the trivial solution (*I*_1_,⋯,*I*_*n*_)^*T*^=**0**, where *T* denotes the transpose of a vector. This implies that *E* ^∗^ does not exist in $\bar {\Omega }$.

Next we show the global asymptotic stability of *E* ^0^. It follows from the Perron-Frobenius theorem (see [38, Theorem 2.1.4]) that *M* ^0^ has a strictly positive left eigenvector (*ℓ*_1_,⋯,*ℓ*_*n*_), *ℓ*_*i*_>0, $i \in {\mathcal {N}}$ corresponding to the eigenvalue *ρ*(*M* ^0^): (*ℓ*_1_,⋯,*ℓ*_*n*_)*ρ*(*M* ^0^)=(*ℓ*_1_,⋯,*ℓ*_*n*_)*M* ^0^. Let $c_{i} : = \ell _{i} /\left (\mu _{i} + \gamma _{i} - Q_{i} \right), \ i \in {\mathcal {N}}$ and $J_{i} (t) : = \int _{t}^{+\infty } \delta _{i}(\xi) \gamma _{i} e^{-\mu _{i} \xi } e^{-\int _{0}^{\xi } \delta _{i}(\eta) \mathrm {d}\eta } \mathrm {d}\xi, \ i \in {\mathcal {N}}$ and consider the following Lyapunov function.

$$\mathcal{L}_{DFE} \left(I_{1},\cdots, I_{n} \right) := \sum_{i=1}^{n} c_{i} \left(I_{i}(t) + \int_{0}^{+\infty} J_{i} (\xi) I_{i} (t-\xi) \mathrm{d} \xi \right). $$

From assumption (A6), *J*_*i*_(*t*)≥0, $i \in {\mathcal {N}}$ for all *t*≥0 and hence, $\mathcal {L}_{DFE} \geq 0$ and the equality holds if and only if (*I*_1_,⋯,*I*_*n*_)≡**0**. Note that

$$\begin{array}{*{20}l} & \left(\int_{0}^{+\infty} J_{i} (\xi) I_{i} (t-\xi) \mathrm{d} \xi \right)' = \int_{0}^{+\infty} J_{i} (\xi) \frac{\partial}{\partial t}I_{i} (t-\xi) \mathrm{d} \xi = -\int_{0}^{+\infty} J_{i} (\xi) \frac{\partial}{\partial \xi}I_{i} (t-\xi) \mathrm{d} \xi \\ & \qquad \quad= -[J_{i} (\xi) I_{i}(t-\xi)]_{0}^{+\infty} + \int_{0}^{+\infty} \frac{\partial}{\partial \xi} J_{i} (\xi) I_{i} (t-\xi) \mathrm{d} \xi \\ & \qquad\quad = Q_{i} I_{i} (t) - \int_{0}^{+\infty} \delta_{i}(\xi) \gamma_{i} I_{i} (t-\xi) e^{-\mu_{i} \xi} e^{-\int_{0}^{\xi} \delta_{i}(\eta) \mathrm{d}\eta} \mathrm{d}\xi, \quad i \in {\mathcal{N}}. \end{array} $$

Hence, the derivative of $\mathcal {L}_{DFE}$ gives

17 $$\begin{array}{*{20}l} \mathcal{L}_{DFE}' &= \sum_{i=1}^{n} c_{i} \left((S_{i}+\sigma_{i} V_{i})\sum\limits_{j=1}^{n}\beta_{ij} \frac{I_{j}}{N_{j}^{*}} -(\mu_{i}+\gamma_{i} - Q_{i}) I_{i} \right)  \\ &= \sum_{i=1}^{n} \ell_{i} \left(\frac{(S_{i} + \sigma_{i} V_{i})\sum\limits_{j=1}^{n}\beta_{ij}I_{j}}{\left(\mu_{i} + \gamma_{i} - Q_{i} \right) N_{j}^{*}} - I_{i} \right)  \\ &= \left(\ell_{1}, \cdots, \ell_{n} \right) \cdot \left(M \left(S_{1},V_{1}, \cdots, S_{n}, V_{n} \right) - E_{n} \right) \cdot (I_{1},\cdots, I_{n})^{T}  \\ &\leq \left(\ell_{1}, \cdots, \ell_{n} \right) \cdot \left(M \left(S_{1}^{0},V_{1}^{0}, \cdots, S_{n}^{0}, V_{n}^{0} \right) - E_{n} \right) \cdot (I_{1},\cdots, I_{n})^{T}  \\ &= \left(\rho (M^{0}) - 1 \right) \left(\ell_{1}, \cdots, \ell_{n} \right) \cdot (I_{1},\cdots, I_{n})^{T} \ \leq \ 0,  \end{array} $$

where *E*_*n*_ denotes the *n*-dimensional unit matrix and · denotes the product of vectors. It is easy to see that when ℜ_0_<1, $\mathcal {L}_{DFE}' = 0$ holds if and only if *I*_*i*_=0 for all $i \in {\mathcal {N}}$, that is, the solution is in the disease-free equilibrium *E* ^0^. When ℜ_0_=1, from the third equality in (17), we see that $\mathcal {L}_{DFE}' = 0$ implies

18 $$\begin{array}{*{20}l} & \left(\ell_{1}, \cdots, \ell_{n} \right) \cdot M\left(S_{1},V_{1},\cdots, S_{n}, V_{n} \right) \cdot (I_{1},\cdots, I_{n})^{T}  \\ & = \left(\ell_{1}, \cdots, \ell_{n} \right) \cdot (I_{1},\cdots, I_{n})^{T}.  \end{array} $$

Suppose that $(S_{1},V_{1},\cdots,S_{n},V_{n}) \neq \left (S_{1}^{0},V_{1}^{0},\cdots,S_{n}^{0},V_{n}^{0}\right)$. Then (*ℓ*_1_,⋯,*ℓ*_*n*_)·*M*(*S*_1_,*V*_1_,⋯,*S*_*n*_,*V*_*n*_)<(*ℓ*_1_,⋯,*ℓ*_*n*_)·*M* ^0^=*ρ*(*M* ^0^)(*ℓ*_1_,⋯,*ℓ*_*n*_)=(*ℓ*_1_,⋯,*ℓ*_*n*_). Hence, (18) has only the trivial solution such that *I*_*i*_=0 for all $i \in {\mathcal {N}}$. This implies that $\mathcal {L}_{DFE}' = 0$ holds only in the disease-free equilibrium $E^{0} \in \bar {\Omega }$. Consequently, from the LaSalle’s invariance principle (see [39]), we can conclude that the disease-free equilibrium *E* ^0^ is globally asymptotically stable.

### Proof of (ii) of Theorem 1

If ℜ_0_>1, then $\left (\ell _{1}, \cdots, \ell _{n} \right) \cdot \left (M \left (S_{1}^{0},V_{1}^{0}, \cdots, S_{n}^{0}, V_{n}^{0} \right) - E_{n} \right) \cdot (I_{1},\cdots, I_{n})^{T} = \left (\rho (M^{0}) - 1 \right) \left (\ell _{1}, \cdots, \ell _{n} \right) \cdot (I_{1},\cdots, I_{n})^{T} \ > \ 0$. Hence, we see from the third equality in (17) that in a neighborhood of $\left (S_{1}^{0},V_{1}^{0},\cdots, S_{n}^{0}, V_{n}^{0}\right)$, $\mathcal {L}_{DFE}' > 0$. This implies the instability of the disease-free equilibrium *E* ^0^.

Since the disease-free equilibrium of *E* ^0^ of system (6) is unstable if ℜ_0_>1, we see from the uniform persistence result of [40] and an argument as in the proof of Proposition 3.3 of [41] that system (6) is uniformly persistent. That is, there exists a positive constant *c*>0 such that for any initial value, it holds that $\liminf _{t\to +\infty } S_{i}(t) \geq c, \ \liminf _{t\to +\infty } V_{i}(t) \geq c$ and $\liminf _{t\to +\infty } I_{i}(t) \geq c$ for all $i \in {\mathcal {N}}$. Since the uniform persistence together with the uniform boundedness implies the existence of an interior equilibrium (see [42, 43]), we see that system (6) has an endemic equilibrium *E* ^∗^∈*Ω*. From (7), we see that the components $\left (S_{1}^{*},V_{1}^{*},I_{1}^{*},\cdots, S_{n}^{*}, V_{n}^{*}, I_{n}^{*}\right)$ of *E* ^∗^ satisfy the following equations.

19 $$\begin{array}{*{20}l} & b_{i} = S_{i}^{*} \sum_{j=1}^{n} \beta_{ij} \frac{I_{j}^{*}}{N_{j}^{*}} + (\mu_{i} + v_{i}) S_{i}^{*} - \omega_{i} V_{i}^{*},  \end{array} $$

20 $$\begin{array}{*{20}l} & v_{i} S_{i}^{*} = \sigma_{i} \sum_{j=1}^{n} \beta_{ij} \frac{I_{j}^{*}}{N_{j}^{*}} + \left(\mu_{i} + \omega_{i} \right) V_{i}^{*},  \end{array} $$

21 $$\begin{array}{*{20}l} & (\mu_{i} + \gamma_{i} - Q_{i}) I_{i}^{*} = (S_{i}^{*} + \sigma_{i} V_{i}^{*}) \sum_{j=1}^{n} \beta_{ij} \frac{I_{j}^{*}}{N_{j}^{*}}, \quad i \in {\mathcal{N}}.  \end{array} $$

As in [14], we define $\theta _{ij} := \left (S_{i}^{*} + \sigma _{i} V_{i}^{*} \right) \beta _{ij} I_{j}^{*}/N_{j}^{*}$, $i, j \in {\mathcal {N}}$ and

$$\Theta := \left(\begin{array}{cccc} \sum\limits_{j\neq 1} \theta_{1j} & -\theta_{21} & \cdots & - \theta_{n1} \\ -\theta_{12} & \sum\limits_{j \neq 2} \theta_{2j} & \cdots & -\theta_{n2} \\ \vdots & \vdots & \ddots & \vdots \\ -\theta_{1n} & -\theta_{2n} & \cdots & \sum\limits_{j \neq n} \theta_{nj} \end{array} \right). $$

Let *φ*:=(*φ*_1_,⋯,*φ*_*n*_)^*T*^ be a basis of the solution space of linear system *Θ* *φ*=0. Then, from [14, Lemma 2.1], we see that the dimension of the solution space is 1 and *φ*_*i*_>0, $i \in {\mathcal {N}}$. In particular, from the form of matrix *Θ*, the following equality holds.

22 $$ \sum_{j=1}^{n} \theta_{ij} \varphi_{i} = \sum_{j=1}^{n} \theta_{ji} \varphi_{j}, \quad i \in {\mathcal{N}}.  $$

Using this *φ* and *H*(*x*):=*x*−1− ln*x*≥*H*(1)=0, we consider the following Lyapunov functional to prove the global asymptotic stability of *E* ^∗^.

$$\begin{array}{*{20}l} & \mathcal{L}_{EE}(S_{1},V_{1},I_{1},\cdots,S_{n},V_{n},I_{n}) := \sum_{i=1}^{n} \varphi_{i} \bigg\{ S_{i}^{*} H\left(\frac{S_{i}}{S_{i}^{*}} \right) +V_{i}^{*} H\left(\frac{V_{i}}{V_{i}^{*}} \right)  \\ & \quad +I_{i}^{*} H\left(\frac{I_{i}}{I_{i}^{*}} \right) + \int_{0}^{+\infty} J_{i} (\xi) I_{i}^{*} H \left(\frac{I_{i} (t-\xi)}{I_{i}^{*}} \right) \mathrm{d}\xi \bigg\}.  \end{array} $$

In order to make this function well-defined, without loss of generality, we can restrict our attention to the solution such that $I_{i}(s)=\varphi _{i} (s), \ i \in \mathcal {N}$ on (−*∞*,0], where *φ*_*i*_(0)=*I*_*i*_(0) and $0 < m_{i} < \varphi _{i}(s) < M_{i} < +\infty, \ s \in (-\infty, 0], \ i \in \mathcal {N}$ for positive constants *m*_*i*_ and *M*_*i*_, $i \in \mathcal {N}$. Then, from the positive invariance of set *Ω* and the uniform persistence of system (6), we see that the Lyapunov functional $\mathcal {L}_{EE}$ is well-defined.

Using (19), we can calculate the derivative of $\mathcal {L}_{EE}$ as follows.

23 $${} \begin{aligned} \mathcal{L}_{EE}' &= \sum_{i=1}^{n} \varphi_{i} \bigg\{ \left(1- \frac{S_{i}^{*}}{S_{i}} \right) \bigg(b_{i}-S_{i}\sum\limits_{j=1}^{n} \beta_{ij}\frac{I_{j}}{N_{j}^{*}}-(\mu_{i}+v_{i})S_{i} +\omega_{i} V_{i} \bigg) \\ &\quad +\left(1- \frac{V_{i}^{*}}{V_{i}} \right) \bigg(v_{i}S_{i} - \sigma_{i} V_{i} \sum\limits_{j=1}^{n}\beta_{ij} \frac{I_{j}}{N_{j}^{*}} - (\mu_{i} +\omega_{i}) V_{i} \bigg) \\ &\quad +\left(1- \frac{I_{i}^{*}}{I_{i}} \right) \bigg((S_{i}+\sigma_{i}V_{i})\sum\limits_{j=1}^{n}\beta_{ij} \frac{I_{j}}{N_{j}^{*}} -(\mu_{i}+\gamma_{i})I_{i} \\ &\quad + \int_{0}^{+\infty} \delta_{i}(\xi) \gamma_{i} I_{i}(t-\xi)e^{-\mu_{i} \xi} e^{-\int_{0}^{\xi}\delta_{i}(\eta) \mathrm{d}\eta} \mathrm{d} \xi \bigg) +\int_{0}^{+\infty} J_{i} (\xi) I_{i}^{*} \frac{\partial}{\partial t} H\left(\frac{I_{i} (t-\xi)}{I_{i}^{*}} \right) \mathrm{d}\xi \bigg\} \\ &= \sum_{i=1}^{n} \varphi_{i} \bigg\{ \left(1- \frac{S_{i}^{*}}{S_{i}} \right) \bigg(S_{i}^{*} \sum_{j=1}^{n} \beta_{ij} \frac{I_{j}^{*}}{N_{j}^{*}} + (\mu_{i} + v_{i}) S_{i}^{*} -\omega_{i} V_{i}^{*} -S_{i}\sum\limits_{j=1}^{n} \beta_{ij}\frac{I_{j}}{N_{j}^{*}} \\ & \quad-(\mu_{i}+v_{i})S_{i} + \omega_{i} V_{i} \bigg) +\left(1- \frac{V_{i}^{*}}{V_{i}} \right) \bigg(\frac{S_{i}}{S_{i}^{*}} v_{i} S_{i}^{*} - \sigma_{i} V_{i} \sum\limits_{j=1}^{n}\beta_{ij} \frac{I_{j}}{N_{j}^{*}} - (\mu_{i} + \omega_{i}) V_{i} \bigg) \\ & \quad+\left(1- \frac{I_{i}^{*}}{I_{i}} \right) \bigg((S_{i}+\sigma_{i}V_{i})\sum\limits_{j=1}^{n}\beta_{ij} \frac{I_{j}}{N_{j}^{*}} -(\mu_{i}+\gamma_{i})I_{i} \\ & \quad+ \int_{0}^{+\infty} \delta_{i}(\xi) \gamma_{i} I_{i}(t-\xi)e^{-\mu_{i} \xi} e^{-\int_{0}^{\xi} \delta_{i}(\eta) \mathrm{d}\eta} \mathrm{d} \xi \bigg) - \int_{0}^{+\infty} J_{i} (\xi) I_{i}^{*} \frac{\partial}{\partial \xi} H\left(\frac{I_{i} (t-\xi)}{I_{i}^{*}} \right) \mathrm{d}\xi \bigg\} \\ &=\sum_{i=1}^{n} \varphi_{i} \bigg\{ \mu_{i} S_{i}^{*} \left(2 - \frac{S_{i}^{*}}{S_{i}} - \frac{S_{i}}{S_{i}^{*}} \right) + v_{i} S_{i}^{*} \left(2 - \frac{S_{i}^{*}}{S_{i}} - \frac{S_{i} V_{i}^{*}}{S_{i}^{*}V_{i}} \right) + \mu_{i} V_{i}^{*} \left(1 - \frac{V_{i}}{V_{i}^{*}} \right) \\ & \quad + \omega_{i} V_{i}^{*} \left(-1 + \frac{S_{i}^{*}}{S_{i}} + \frac{V_{i}}{V_{i}^{*}} -\frac{S_{i}^{*} V_{i}}{S_{i}V_{i}^{*}}+1-\frac{V_{i}}{V_{i}^{*}} \right) \\ & \quad+ S_{i}^{*} \sum_{j=1}^{n} \beta_{ij} \frac{I_{j}^{*}}{N_{j}^{*}} \left(1-\frac{S_{i}^{*}}{S_{i}} \right) + \left(S_{i}^{*} + \sigma_{i} V_{i}^{*} \right) \sum_{j=1}^{n} \beta_{ij} \frac{I_{i}}{N_{j}^{*}} - (S_{i}+\sigma_{i}V_{i})\sum\limits_{j=1}^{n}\beta_{ij} \frac{I_{j}^{*}}{N_{j}^{*}} \frac{I_{i}^{*} I_{j}}{I_{i} I_{j}^{*}} \\ & \quad+(\mu_{i}+\gamma_{i}) I_{i}^{*} \left(1-\frac{I_{i}}{I_{i}^{*}} \right) + \left(1-\frac{I_{i}}{I_{i}^{*}} \right) \int_{0}^{+\infty} \delta_{i}(\xi) \gamma_{i} I_{i}(t-\xi)e^{-\mu_{i} \xi} e^{-\int_{0}^{\xi} \delta_{i}(\eta) \mathrm{d}\eta} \mathrm{d} \xi \bigg) \\ & \quad- \int_{0}^{+\infty} J_{i} (\xi) I_{i}^{*} \frac{\partial}{\partial \xi} H\left(\frac{I_{i} (t-\xi)}{I_{i}^{*}} \right) \mathrm{d}\xi \bigg\}.  \end{aligned}  $$

Now it follows from integration by parts that

$$\begin{array}{*{20}l} &\int_{0}^{+\infty} J_{i} (\xi) I_{i}^{*} \frac{\partial}{\partial \xi} H\left(\frac{I_{i} (t-\xi)}{I_{i}^{*}} \right) \mathrm{d}\xi  \\ &= -J_{i}(0) I_{i}^{*} H\left(\frac{I_{i}}{I_{i}^{*}} \right) + \int_{0}^{+\infty} \delta_{i}(\xi) \gamma_{i} e^{-\mu_{i} \xi} e^{-\int_{0}^{\xi} \delta_{i}(\eta) \mathrm{d}\eta} I_{i}^{*} H\left(\frac{I_{i}(t-\xi)}{I_{i}^{*}}\right) \mathrm{d}\xi  \\ & = -Q_{i} \left(I_{i} - I_{i}^{*} -I_{i}^{*} \ln \frac{I_{i}(t)}{I_{i}^{*}} \right)  \\ &\qquad+ \int_{0}^{+\infty} \delta_{i}(\xi) \gamma_{i} e^{-\mu_{i} \xi} e^{-\int_{0}^{\xi} \delta_{i}(\eta) \mathrm{d}\eta} \left(I_{i}(t-\xi) - I_{i}^{*} -I_{i}^{*} \ln \frac{I_{i}(t-\xi)}{I_{i}^{*}} \right) \mathrm{d}\xi.  \end{array} $$

Hence, (23) can be calculated as follows:

24 $$\begin{array}{*{20}l} &  \mathcal{L}_{EE}'  \\ &  = \sum_{i=1}^{n} \varphi_{i} \bigg\{ \mu_{i} S_{i}^{*} \left(2 - \frac{S_{i}^{*}}{S_{i}} - \frac{S_{i}}{S_{i}^{*}} \right) + v_{i} S_{i}^{*} \left(2 - \frac{S_{i}^{*}}{S_{i}} - \frac{S_{i} V_{i}^{*}}{S_{i}^{*}V_{i}} \right) + \mu_{i} V_{i}^{*} \left(1 - \frac{V_{i}}{V_{i}^{*}} \right)  \\ & + \omega_{i} V_{i}^{*} \left(\frac{S_{i}^{*}}{S_{i}} -\frac{S_{i}^{*} V_{i}}{S_{i}V_{i}^{*}} \right) + S_{i}^{*} \sum_{j=1}^{n} \beta_{ij} \frac{I_{j}^{*}}{N_{j}^{*}} \left(1-\frac{S_{i}^{*}}{S_{i}} \right) + \left(S_{i}^{*} + \sigma_{i} V_{i}^{*} \right) \sum_{j=1}^{n} \beta_{ij} \frac{I_{i}}{N_{j}^{*}}  \\ & - (S_{i}+\sigma_{i}V_{i})\sum\limits_{j=1}^{n}\beta_{ij} \frac{I_{j}^{*}}{N_{j}^{*}} \frac{I_{i}^{*} I_{j}}{I_{i} I_{j}^{*}} +(\mu_{i}+\gamma_{i} - Q_{i}) I_{i}^{*} \left(1-\frac{I_{i}}{I_{i}^{*}} \right) - Q_{i} I_{i}^{*} \ln \frac{I_{i}}{I_{i}^{*}}  \\ &-I_{i}^{*} \int_{0}^{+\infty} \delta_{i}(\xi) \gamma_{i} \left(\frac{I_{i}(t-\xi)}{I_{i}} - 1 - \ln \frac{I_{i}(t-\xi)}{I_{i}^{*}} \right) e^{-\mu_{i} \xi} e^{-\int_{0}^{\xi} \delta_{i}(\eta) \mathrm{d}\eta} \mathrm{d} \xi \bigg\}.  \end{array} $$

From (21) and (22), we have

25 $$\begin{array}{*{20}l} & \sum_{i=1}^{n} \varphi_{i} \left(\mu_{i} + \gamma_{i} - Q_{i} \right) I_{i} =\sum_{i=1}^{n} \varphi_{i} \left(\mu_{i} + \gamma_{i} - Q_{i} \right) I_{i}^{*} \frac{I_{i}}{I_{i}^{*}}  \\ &=\sum_{i=1}^{n} \varphi_{i} \left(S_{i}^{*} + \sigma_{i} V_{i}^{*} \right) \sum_{j=1}^{n} \beta_{ij} \frac{I_{j}^{*}}{N_{j}^{*}} \frac{I_{i}}{I_{i}^{*}}  \\ &= \sum_{i=1}^{n} \frac{I_{i}}{I_{i}^{*}} \sum_{j=1}^{n} \theta_{ij} \varphi_{i} = \sum_{i=1}^{n} \frac{I_{i}}{I_{i}^{*}} \sum_{j=1}^{n} \theta_{ji} \varphi_{j} = \sum_{i=1}^{n} \sum_{j=1}^{n} \theta_{ji} \frac{I_{i}}{I_{i}^{*}} \varphi_{j}  \\ & = \sum_{i=1}^{n} \sum_{j=1}^{n} \theta_{ij} \frac{I_{j}}{I_{j}^{*}} \varphi_{i} = \sum_{i=1}^{n} \varphi_{i} \sum_{j=1}^{n} \theta_{ij} \frac{I_{j}}{I_{j}^{*}}= \sum_{i=1}^{n} \varphi_{i} \left(S_{i}^{*} + \sigma_{i} V_{i}^{*} \right) \sum_{j=1}^{n} \beta_{ij} \frac{I_{j}}{N_{j}^{*}}.  \end{array} $$

Hence, using (20), (21) and (25), we can calculate (24) as follows:

26 $$\begin{array}{*{20}l}  \mathcal{L}_{EE}' &= \sum_{i=1}^{n} \varphi_{i} \bigg\{ \mu_{i} S_{i}^{*} \left(2 - \frac{S_{i}^{*}}{S_{i}} - \frac{S_{i}}{S_{i}^{*}} \right) + \mu_{i} V_{i}^{*} \left(3 - \frac{S_{i}^{*}}{S_{i}} - \frac{S_{i} V_{i}^{*}}{S_{i}^{*}V_{i}} - \frac{V_{i}}{V_{i}^{*}} \right)  \\ &\quad +\omega_{i} V_{i}^{*} \left(2- \frac{S_{i}V_{i}^{*}}{S_{i}^{*} V_{i}} -\frac{S_{i}^{*} V_{i}}{S_{i} V_{i}^{*}} \right) + S_{i}^{*} \sum_{j=1}^{n} \beta_{ij} \frac{I_{j}^{*}}{N_{j}^{*}} \left(2-\frac{S_{i}^{*}}{S_{i}} - \frac{S_{i} I_{i}^{*} I_{j}}{S_{i}^{*} I_{i} I_{j}^{*}} \right)  \\ &\quad + \sigma_{i} V_{i}^{*} \sum_{j=1}^{n} \beta_{ij} \frac{I_{j}^{*}}{N_{j}^{*}} \left(3 - \frac{S_{i}^{*}}{S_{i}} - \frac{S_{i} V_{i}^{*}}{S_{i}^{*}V_{i}} - \frac{V_{i} I_{i}^{*} I_{j}}{V_{i}^{*} I_{i} I_{j}^{*}} \right)  \\ &\quad - Q_{i} I_{i}^{*} \ln \frac{I_{i}}{I_{i}^{*}} + Q_{i} I_{i}^{*} \ln \frac{I_{i}}{I_{i}^{*}} -I_{i}^{*} \int_{0}^{+\infty} \delta_{i}(\xi) \gamma_{i} H \left(\frac{I_{i}(t-\xi)}{I_{i}}\right) e^{-\mu_{i} \xi} e^{-\int_{0}^{\xi} \delta_{i}(\eta) \mathrm{d}\eta} \mathrm{d} \xi \bigg\}.  \end{array} $$

Using the inequality of arithmetic and geometric means, we see that the first three terms in the right-hand side of (26) are non-positive and equal to zero if and only if $\left (S_{i}, V_{i} \right) = \left (S_{i}^{*}, V_{i}^{*} \right)$, $i \in {\mathcal {N}}$. From the positivity of the function *H*(*x*), we see that the last term in the right-hand side of (26) is non-positive. Hence, taking the maximum as in [10] and using the graph-theoretic approach as in [14], we can evaluate (26) as follows:

27 $$\begin{array}{*{20}l}  \mathcal{L}_{EE}' &\leq \sum_{i=1}^{n} \varphi_{i} \sum_{j=1}^{n} \left(S_{i}^{*} + \sigma_{i} V_{i}^{*} \right) \beta_{ij} \frac{I_{j}^{*}}{N_{j}^{*}}  \\ & \quad \times \max \left(2- \frac{S_{i}^{*}}{S_{i}} - \frac{S_{i} I_{i}^{*} I_{j}}{S_{i}^{*} I_{i} I_{j}^{*}}, 3 - \frac{S_{i}^{*}}{S_{i}} - \frac{S_{i} V_{i}^{*}}{S_{i}^{*} V_{i}} - \frac{V_{i} I_{i}^{*} I_{j}}{V_{i}^{*} I_{i} I_{j}^{*}} \right)  \\ &= \sum_{i=1}^{n} \varphi_{i} \sum_{j=1}^{n} \theta_{ij} \max \left(2- \frac{S_{i}^{*}}{S_{i}} - \frac{S_{i} I_{i}^{*} I_{j}}{S_{i}^{*} I_{i} I_{j}^{*}}, 3 - \frac{S_{i}^{*}}{S_{i}} - \frac{S_{i} V_{i}^{*}}{S_{i}^{*} V_{i}} - \frac{V_{i} I_{i}^{*} I_{j}}{V_{i}^{*} I_{i} I_{j}^{*}} \right)  \\ &= \sum_{G \in \Gamma} w (G) \sum_{(i,j) \in A(CG)} \max \left(2- \frac{S_{i}^{*}}{S_{i}} - \frac{S_{i} I_{i}^{*} I_{j}}{S_{i}^{*} I_{i} I_{j}^{*}}, 3 - \frac{S_{i}^{*}}{S_{i}} - \frac{S_{i} V_{i}^{*}}{S_{i}^{*} V_{i}} - \frac{V_{i} I_{i}^{*} I_{j}}{V_{i}^{*} I_{i} I_{j}^{*}} \right),  \end{array} $$

where *Γ* denotes the set of all unicyclic graphs included in directed graphs with vertices {1,2,⋯,*n*}, *G* denotes the unicyclic graph included in *Γ*, *w*(*G*) denotes the weight of graph *G*, *CG* denotes the unicycle included in *G* and *A*(*C* *G*) denotes the set of all arcs included in *CG*. For instance, for a unicycle *C* *G*:1→2→1, we have *A*(*C* *G*)={(1,2),(2,1)} and thus,

$$\begin{array}{*{20}l} & \sum_{(i,j) \in A(CG)} \max \left(2- \frac{S_{i}^{*}}{S_{i}} - \frac{S_{i} I_{i}^{*} I_{j}}{S_{i}^{*} I_{i} I_{j}^{*}}, 3 - \frac{S_{i}^{*}}{S_{i}} - \frac{S_{i} V_{i}^{*}}{S_{i}^{*} V_{i}} - \frac{V_{i} I_{i}^{*} I_{j}}{V_{i}^{*} I_{i} I_{j}^{*}} \right) \\ &= \max \left(2- \frac{S_{1}^{*}}{S_{1}} -\frac{S_{1} I_{1}^{*} I_{2}}{S_{1}^{*} I_{1} I_{2}^{*}}, \ 3- \frac{S_{1}^{*}}{S_{1}} - \frac{V_{1}^{*} S_{1}}{V_{1} S_{1}^{*}} - \frac{V_{1} I_{1}^{*} I_{2}}{V_{1}^{*} I_{1} I_{2}^{*}} \right) \\ &\quad + \max \left(2- \frac{S_{2}^{*}}{S_{2}} -\frac{S_{2} I_{2}^{*} I_{1}}{S_{2}^{*} I_{2} I_{1}^{*}}, \ 3- \frac{S_{2}^{*}}{S_{2}} - \frac{V_{2}^{*} S_{2}}{V_{2} S_{2}^{*}} - \frac{V_{2} I_{2}^{*} I_{1}}{V_{2}^{*} I_{2} I_{1}^{*}} \right) \\ &= \max \left(4- \frac{S_{1}^{*}}{S_{1}} -\frac{S_{1} I_{1}^{*} I_{2}}{S_{1}^{*} I_{1} I_{2}^{*}} - \frac{S_{2}^{*}}{S_{2}} -\frac{S_{2} I_{2}^{*} I_{1}}{S_{2}^{*} I_{2} I_{1}^{*}}, \right. \\ &\hspace{2.5em} \left. 5- \frac{S_{1}^{*}}{S_{1}} -\frac{S_{1} I_{1}^{*} I_{2}}{S_{1}^{*} I_{1} I_{2}^{*}} - \frac{S_{2}^{*}}{S_{2}} - \frac{V_{2}^{*} S_{2}}{V_{2} S_{2}^{*}} - \frac{V_{2} I_{2}^{*} I_{1}}{V_{2}^{*} I_{2} I_{1}^{*}}, \right. \\ & \hspace{2.5em} \left. 5 - \frac{S_{1}^{*}}{S_{1}} - \frac{V_{1}^{*} S_{1}}{V_{1} S_{1}^{*}} - \frac{V_{1} I_{1}^{*} I_{2}}{V_{1}^{*} I_{1} I_{2}^{*}} - \frac{S_{2}^{*}}{S_{2}} -\frac{S_{2} I_{2}^{*} I_{1}}{S_{2}^{*} I_{2} I_{1}^{*}}, \right. \\ & \hspace{2.5em} \left. 6 -\frac{S_{1}^{*}}{S_{1}} - \frac{V_{1}^{*} S_{1}}{V_{1} S_{1}^{*}} - \frac{V_{1} I_{1}^{*} I_{2}}{V_{1}^{*} I_{1} I_{2}^{*}} - \frac{S_{2}^{*}}{S_{2}} - \frac{V_{2}^{*} S_{2}}{V_{2} S_{2}^{*}} - \frac{V_{2} I_{2}^{*} I_{1}}{V_{2}^{*} I_{2} I_{1}^{*}} \right). \end{array} $$

We see that all elements in the max in the last expression of the above formula are non positive because of the inequality of arithmetic and geometric means. Similarly, we can easily check that for all unicycles *CG* with at most *n* vertices, the second sum in the last expression of (27) are non-positive (see [10, Proof of Theorem 4.1]). Hence, $\mathcal {L}_{EE}'$ is non-positive and it is easy to check that the equality $\mathcal {L}_{EE}' = 0$ holds if and only if $\left (S_{1},V_{1},I_{1},\cdots, S_{n},V_{n},I_{n} \right) = \left (S_{1}^{*},V_{1}^{*},I_{1}^{*},\cdots, S_{n}^{*},V_{n}^{*},I_{n}^{*} \right) $. This implies, from the LaSalle’s invariance principle, that the endemic equilibrium *E* ^∗^ is globally asymptotically stable.
